# Molecular adsorbent recirculating system (MARS) in acute liver injury and graft dysfunction: Results from a case-control study

**DOI:** 10.1371/journal.pone.0175529

**Published:** 2017-04-12

**Authors:** Hans U. Gerth, Michele Pohlen, Gerold Thölking, Hermann Pavenstädt, Marcus Brand, Christian Wilms, Anna Hüsing-Kabar, Dennis Görlich, Iyad Kabar, Hartmut H. J. Schmidt

**Affiliations:** 1 Department of Medicine D, Division of General Internal Medicine, Nephrology, and Rheumatology, University Hospital Muenster, Muenster, Germany; 2 Department of Medicine A, Hematology and Oncology, University Hospital Muenster, Muenster, Germany; 3 Department of Transplant Medicine, University Hospital Muenster, Muenster, Germany; 4 Institute of Biostatistics and Clinical Research, University Muenster, Muenster, Germany; Texas A&M University, UNITED STATES

## Abstract

**Background:**

The primary therapeutic goals in the treatment of liver injury are to support liver regeneration or bridge the gap to liver transplantation (LT). Molecular adsorbent recirculating system (MARS) therapy has shown beneficial effects for specific symptoms of liver failure; however, general survival advantages have not yet been demonstrated.

**Aim:**

We studied the effects of MARS therapy compared to standard medical treatment (SMT) in two patient cohorts: in patients with an acute liver injury and in those with graft dysfunction (GD).

**Methods:**

We report on our experience over a 6.5-year period with 73 patients treated with SMT or with SMT and MARS (MARS group). In total, 53 patients suffered from acute liver injury in their native liver without a preexisting liver disease (SMT: n = 31, MARS: n = 22), and 20 patients showed a severe GD after LT (SMT: n = 10, MARS: n = 10).

**Results:**

The entire cohort was predominantly characterized by hemodynamically and respiratorily stable patients with a low hepatic encephalopathy (HE) grade and a model of end-stage liver disease (MELD) score of 20.57 (MARS) or 22.51 (SMT, p = 0.555). Within the MARS group, the median number of extracorporeal therapy sessions was four (range = 3–5 sessions). Independent of the underlying etiology, MARS improved the patients’ bilirubin values in the short term compared to SMT alone. In patients with acute liver injury, this response was sustained even after the end of MARS therapy. By contrast, the majority of patients with GD and an initial response to MARS therapy experienced worsened hyperbilirubinemia. No differences in 28-day mortality were observed with respect to acute liver injury (MARS 5.3% (95% CI: 0–15.3); SMT 3.3% (95% CI: 0–9.8), p = 0.754) or GD (MARS 20.0% (95% CI: 0–44.7), SMT 11.1% (95% CI: 0–31.7), p = 0.478).

**Conclusions:**

Although it did not improve 28-day mortality, MARS therapy improved the short-term response in patients with acute liver injury as well as in those with GD. In cases of acute hepatic injury, the use of MARS therapy resulted in the sustained stabilization of liver function and improved liver regeneration. A short-term response to MARS may predict the future course of the disease.

## Introduction

The various forms of severe liver damage have different causative pathomechanisms and thus require specific therapies. The spectrum varies from acute liver injury and its transition into acute liver failure (ALF) to acute-on-chronic liver failure (ACLF) in patients with preexisting liver disease and even to graft dysfunction (GD) after liver transplantation (LT) [1,2]. Regardless of the underlying etiology, the primary common therapeutic goal is to support the liver in its regeneration or to bridge the time until LT.

The possibility of replacing at least some liver function by an artificial liver support (ALS) system that can be applied additively or alternatively to LT remains very important. Among these systems, molecular adsorbent recirculating system (MARS) therapy represents one of the most studied methods with a proven beneficial effect on hepatic encephalopathy (HE), hepatorenal syndrome (HRS) and hyperbilirubinemia [3–5]; however, the mortality-related results of these studies are controversial. A general survival advantage of any ALS therapy for liver failure has not yet been shown and is restricted to meta-analyses or patient subgroups [6–8].

One large trial evaluated the use of MARS among patients with ACLF but failed to demonstrate a reduction in short-term mortality [9]. Additionally, another randomized controlled trial analyzing the same ALS system in ALF patients did not show any survival benefit [10]. Notably, in the latter study, many patients in the therapy arm were already successfully transplanted before MARS dialysis was performed. Additionally, the median cumulative duration of therapy was only 10 hours. However, patients who received several dialyses (≥ 3) exhibited significantly prolonged transplant-free survival. This positive association between the number of MARS sessions and survival was confirmed by several other studies [11,12].

Although ALS may not improve overall survival in general, specific patient subgroups may benefit [13]. MARS is the most extensively studied ALS modality; however, data on its use in GD are limited [14,15]. Therefore, the appropriate indications for MARS therapy and clear criteria for patient selection remain unknown. We sought to identify the patient subgroups that would benefit from MARS treatment. Thus, we generated new hypotheses on the optimal patient selection for MARS therapy and developed improved management strategies for before and after LT.

Here, we report on our experience over a 6.5-year period with a total of 73 patients. Overall, 53 patients suffered from acute liver injury in their native liver without preexisting liver disease, and 20 patients had a severe GD after LT. The decision to initiate MARS therapy was made on a case-by-case basis, and patients not treated with MARS therapy served as a control cohort.

## Patients and methods

This study was based on patients treated at the University Hospital Muenster (a quaternary care academic hospital in Germany) from January 2009 to July 2015.

### Patient selection

All patients with evidence of liver disease leading to hospital admission were retrospectively identified using the German modification of the International Statistical Classification of Diseases and Related Health Problems (ICD-10-GM).

To focus on patients with acute liver injury and GD, patients with a preexisting liver disease and acute decompensation (AD) or ACLF were excluded. All patients classified as having acute liver injury had no medical history of liver disorders or signs of chronic liver disease in clinical and ultrasound examination. In addition, patients with cancer, hemolysis, or cardiac disease causing liver failure and those with infection and/or extrahepatic jaundice were excluded from the analysis.

The selection process did not identify children (age < 18 years), HIV-positive patients, pregnant/breastfeeding women, patients with severe thrombocytopenia (platelet count < 50,000/μL) before initiating therapy, those with clinical evidence of disseminated intravascular coagulation (DIC) or those with active bleeding; thus, these groups were not excluded from the analysis.

At our hospital, MARS was predominantly applied to hemodynamically and respiratorily stable patients (no need of vasopressors or ventilatory support) with severe liver impairment (bilirubin values ≥ 6 mg/dL) independent of additional organ failures secondary to the liver (MARS group). Subsequently, all patients fulfilling the same conditions with the exception of the application of MARS were classified as the control group (SMT group).

Complimentary applied therapies and supportive care were provided to both groups according to the corresponding institutional guidelines.

All patients provided written informed consent prior to the initiation of medical treatment. Approval for this investigation conforming to the ethical guidelines of the 1975 Declaration of Helsinki was obtained from the Ethics Board of the Westphalian Wilhelms-University Muenster and the Physicians Chamber of Westphalia-Lippe, Germany (Reference Number: 2015-725-f-S).

### Standard medical treatment—SMT

All patients were treated in a specialized liver unit according to national guidelines. In detail, coagulation support was not routinely offered. Coagulation factor substitution was performed only in case of hemorrhage or disseminated intravascular coagulation. N-Acetylcysteine was given to all patients irrespective of etiology for a maximum of five days. Prophylaxis or empirical treatment with antibiotics and antifungals were used according to in-house treatment recommendations.

HE was graded by adapting the West Haven Criteria, and the Model of End-Stage Liver Disease (MELD) score was calculated according to Kamath et al. [16–18].

Patients with severe liver failure were listed for LT in accordance with the criteria of the Eurotransplant International Foundation. Regular diagnostic patient screens included serological tests, hepatitis serology, autoimmune markers, ultrasound and/or CT imaging and clinical examination.

### Extracorporeal treatment—MARS

In the MARS group, was applied additionally to SMT. With the exception of this extracorporeal treatment, therapy did not differ between both groups.

MARS therapy was performed almost daily (at least three procedures within five days) using a Fresenius 5008 dialysis machine (Fresenius Medical Care Deutschland GmbH, Bad Homburg, Germany) and a MARS Monitor (Gambro Lundia AB, Lund, Sweden). The corresponding treatment time was 360 minutes on average. The MARS Treatment Kit, type 1412/1 (Gambro Lundia AB, Lund, Sweden) was used for MARS therapy. The albumin circuit contained 500 mL of 20% human albumin. Blood access was established through a conventional double lumen hemodialysis catheter via the patient’s jugular or femoral veins.

Blood flow rates were 250–350 mL/min, and the flow rates in the albumin circuit were approximately 250 mL/min. If not contraindicated, heparin was used for anticoagulation. Ultrafiltration was adjusted to control volume balance when necessary. For safety, the patient’s blood pressure and heart rate were continuously monitored during the MARS sessions, and laboratory measurements were taken at least once per day. The platelet count was monitored closely, and any platelet count below 50,000/μL resulted in the discontinuation of MARS.

### Outcome

The outcome parameters on day 28 after the initiation of therapy were defined as follows: liver (re)transplantation, mortality (death due to any cause) or liver transplantation-free survival (censoring patients if it was the date of their LT or if they were known to be alive at the time of the last follow-up assessment). Patients who did not suffer from any event (death or LT) within the follow-up period were censored at day 28. The calculations were performed not only for the entire cohort but also for subgroups of patients with acute liver injury or a history of LT.

The changes in important blood values (bilirubin, creatinine, international normalized ratio [INR], platelets, hemoglobin, and serum sodium) were examined in the short-term course (4 days) in both therapy cohorts (SMT *vs*. MARS treatment). Response to therapy was assessed by analyzing the dynamics of the bilirubin values. This assessment resulted in the following classification: An increase or minimal reduction (<5%) within this short-term course (4 days) was assessed as no response (NR). A moderate decrease of 5–25% was described as partial response (PR). In addition, a decline of 25% or more was considered as therapy response (R). This tripartitioning (NR *vs*. PR *vs*. R) was used for the comparison of not only both therapeutic modalities (SMT *vs*. MARS) but also both etiologies (acute liver injury *vs*. GD). To assess the sustainability of therapy response, the same classification was used again to evaluate the response two weeks after the termination of MARS therapy (patients with liver transplantation or missing data at the follow-up or those who had died were excluded).

### Data collection and statistics

Clinical and laboratory data were obtained from the patients’ electronic medical records.

Data are presented as absolute numbers, percentages, and means with their corresponding standard deviations (SD); missing data were replaced by mean values. Differences between the MARS and SMT group were analyzed using a two-sided Mann-Whitney-U test in the case of continuous variables. The two-sided chi-squared test was used to compare categorical variables.

Repeatedly measured laboratory parameters were logarithmized (log_10_) before analysis. Linear mixed models (GLMM), assuming normally distributed data, have been used to analyze the time course of bilirubin, as main indicator for treatment response. Data of the first seven days after therapy start was used to assess the initial response. The GLMM includes fixed effect for time (in days), group (MARS vs SMT) and the interaction time*group. Repeated measurements are modelled using a random intercept per subject (i.e. patients). Time is modelled as continuous variable and correlations between time points were modelled by a spatial power model (cov(i,j) = ϭ^2^ρ^dij^). The model was fitted using an empirical estimator to be robust against initial model misspecification. Marginal model predictions were used to predict the overall mean time course estimated in the model. Estimated slopes (absolute bilirubin change per day) and 95% confidence intervals are reported on the original scale. The two subgroups (acute liver failure and graft dysfunction) were modelled separately.

Liver-transplant-free survival was estimated using the Kaplan-Meier method. Additionally, the absolute and relative frequency of events (censoring, LT, and death) are reported.

Two-tailed p-values ≤ 0.05 were considered significant. The data was analyzed within an exploratory approach, no adjustment for the multiple comparison problem has been made. Statistical analyses were performed using IBM SPSS Statistics for Windows, version 22.0 (IBM Corp., Armonk, NY, USA) and GraphPad Prism 5 for Windows, version 5.01 (GraphPad Software, La Jolla, CA, USA). Generalized linear mixed models were fitted using SAS/STAT software's capabilities (Version 14.1 of the SAS Software 9.4, SAS Institute Inc., Cary, NC, USA).

## Results

### Study population

**Fig 1:**
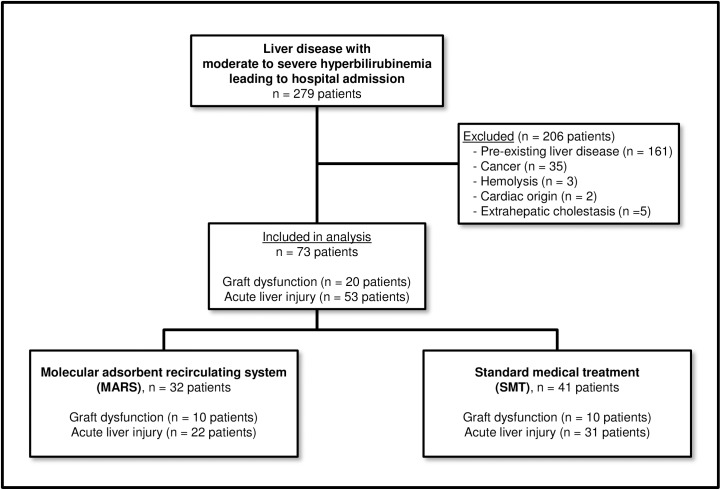
Flow chart displaying the selection procedure and patient numbers. All patients with moderate to severe hyperbilirubinemia (≥ 6 mg/dL) leading to a hospital admission between January 2009 and June 2015 were screened for acute liver injury or graft dysfunction. All patients in the final cohort received either SMT (41 patients) or SMT and MARS (32 patients).

A total of 279 patients with liver disease leading to hospital admission and jaundice (hyperbilirubinemia ≥6 mg/dL) were identified through an in-house database search according to ICD and OPS codes. The search criteria and subsequent patient selection procedure are depicted in **Fig 1**. Patients with liver failure due to a cause other than acute liver injury (without preexisting liver disease) or graft dysfunction were excluded. Of the evaluated patients, 73 met the eligibility criteria and were included in the study. A total of 41 patients received SMT exclusively, and 32 patients were treated with SMT and MARS. In the two groups, 53 patients had acute liver injury and 20 patients suffered from graft dysfunction after LT.

**Table 1** displays the baseline patient criteria in detail. The predominant etiology of liver disease was non-paracetamol hepatitis (drug induced liver injury) (n = 50, 68%).

**Table 1:** Baseline patient characteristics: Comparison of MARS and standard medical treatment (SMT).

Laboratory Parameter			MARS (n = 32)	SMT (n = 41)	p-value
**Age (years)**	mean (SD)		48.9 (17.8)	49.4 (16.0)	0.925
	range		18–76	21–85	
**Male sex, n (%)**			20 (62.5)	23 (56.1)	0.581
**Body weight (kg), mean (SD)**			82.1 (18.1)	75.9 (21.5)	0.146
**Etiology of liver disease, n (%)**	Paracetamol		2 (6.2)	1 (2.4)	
	Non-paracetamol		20 (62.5)	30 (73.2)	
		Viral	1	7	
		Autoimmune	0	9	
		Drug-induced	15	12	
		Mushroom poisoning/toxin	0	0	
		Unknown/other	4	2	
	Graft dysfunction		10 (31.3)	10 (24.4)	
		Rejection	5	2	
		Biliary casts	2	3	
		Unknown/other	3	5	
**Hepatic encephalopathy, n (%)**	Grade ≤1		32 (100)	38 (92.7)	0.417
	Grade 2–3		0	3 (7.3)	
**Mechanical ventilation, n**			0	0	n.a.
**MAP (mmHg)**			97.6 (11.5)	98.6 (14.8)	0.804
	Vasopressors, n		0	0	n.a.
**Heart rate (bpm)**			75.9 (8.6)	82.3 (14.9)	0.206
**Laboratory data, mean (SD)**	Total bilirubin (mg/dL)		18.8 (9.3)	14.9 (8.0)	**0.037**
	AST (U/L)		570.5 (721.2)	1079.1 (915.1)	**0.011**
	ALT (U/L)		826.7 (703.7)	1414.5 (1298.4)	0.058
	Serum sodium (mEq/L)		136.5 (3.8)	138.2 (3.8)	0.127
	Serum potassium (mEq/L)		4.0 (4.9)	3.9 (0.5)	0.333
	Creatinine (mg/dL)		1.2 (0.6)	1.3 (1.2)	0.740
	BUN (mg/dL)		24.8 (18.1)	20.0 (13.9)	0.235
	White blood count (10^3^ cells/μL)		7.7 (3.3)	7.2 (2.96)	0.687
	Hemoglobin (g/dL)		11.9 (2.2)	12.8 (2.8)	0.055
	Platelets (10^3^ cells/μL)		229.8 (109.9)	182.4 (92.8)	0.074
	Albumin (g/dL)		3.4 (0.5)	3.2 (0.5)	0.768
	Prothrombin level (Quick) (%)		72.9 (26.8)	56.4 (20.5)	**0.012**
	INR		1.3 (0.3)	1.5 (0.5)	**0.015**
**MELD Score, mean (SD)**			20.6 (7.1)	22.5 (4.8)	0.555

Abbr.: MAP: Mean arterial pressure; BUN: blood urea nitrogen; INR: international normalized ratio; MELD: model of end-stage liver disease; SD: standard deviation; AST: aspartate transaminase; ALT: alanine transaminase

Collectively, the analyzed patient cohort was characterized by predominantly hemodynamically and respiratorily stable patients who had a low HE grade (n = 70, 95%) if they had one at all.

Patients in the MARS group had initial bilirubin values with a mean of 18.85 mg/dL (SD: 9.3 mg/dL), which was higher than the mean of 14.89 mg/dL (SD: 8.0 mg/dL) in the SMT group (p = 0.037). Furthermore, the transaminase levels and INR values were increased compared to the SMT group. However, the MELD scores did not significantly differ between both groups (MARS: 20.57 vs. SMT: 22.51, p = 0.555).

A more differentiated presentation of the baseline laboratory values with respect to the etiology is presented in **S1 Table**. Here, patients after LT (GD group) treated with SMT had lower bilirubin values but higher INR values than those treated with MARS. The time period from LT to GD was nearly equal in the two subgroups (MARS: median of 24.4 months *vs*. SMT: median of 23.2 months).

In patients afflicted with an acute liver injury, transaminase levels in the SMT group were markedly higher than those in the MARS patients. However, most clinical and laboratory parameters, including MELD scores, were approximately balanced in the two subgroups.

### Response and outcome

Within the MARS group, the median number of extracorporeal therapy sessions was four (range = 3–5 sessions), each consisting of six hours of treatment time (**Table 2**).

**Table 2:** Treatment and outcome data: Comparison of MARS and standard medical treatment (SMT).

Laboratory Parameter			MARS (n = 32)	SMT (n = 41)	p-value
**Number of therapy sessions, median (IQR)**			4 (3–5)	-	-
**Treatment time (hours), median (IQR)**			6 (6–6)	-	**-**
**Average blood flow (ml/min), median (IQR)**			200 (187–250)	-	**-**
**Outcome on day 28, n (%)**	All patients (n = 73)	survival	25 (78.1)	37 (90.2)	0.348
		LT	4 (12.5)	2 (4.9)	
		mortality	3 (9.4)	2 (4.9)	
	Acute liver injury (n = 53)	survival	19 (86.4)	29 (93.5)	0.633
		LT	2 (9.1)	1 (3.2)	
		mortality	1 (4.5)	1 (3.2)	
	Graft dysfunction (n = 20)	survival	6 (60)	8 (80)	0.621
		LT	2 (20)	1 (10)	
		mortality	2 (20)	1 (10)	

Abbr.: IQR: interquartile range Q1 –Q3; LT: liver transplantation

When analyzing the mortality or LT-free survival rate on day 28, no significant differences between SMT and MARS were observed in the entire cohort or after splitting by etiology. In particular, there was a slight trend toward increased 28-day mortality in both subgroups after MARS treatment compared to SMT: 28-day mortality in patients with acute liver injury (MARS 5.3% (95% CI: 0–15.3%); SMT 3.3% (95% CI: 0–9.8), p = 0.754) and in those with GD (MARS 20.0% (95% CI: 0–44.7), SMT 11.1% (95% CI: 0–31.7), p = 0.478).

In the MARS group, two patients died due to infection/multiorgan failure, and one patient died due to bleeding. In the SMT group, one patient died during surgery because of hemodynamic instability; another patient’s death was infection/multiorgan failure-related. However, the number of transplanted patients in the MARS group was higher than in the SMT group (MARS 4 patients, SMT 2 patients, p = 0.239).

A detailed description of the changes in important laboratory values within four days can be found in **Table 3**. With respect to bilirubin values, a significant reduction could be observed after MARS therapy and in subgroup analyses of both etiologies (**S2 Table**).

**Table 3:** Short-term response of laboratory parameters.

Laboratory Parameter		MARS	SMT	p-value
**Bilirubin (mg/dL)**	Baseline (mean, SD)	18.8 (9.3)	14.9 (8.0)	
	Day 4 (mean, SD)	14.6 (8.0)	14.4 (7.8)	
	Percentage of change (from baseline value, %)	-22.3	-3.4	**0.001**
**Creatinine (mg/dL)**	Baseline (mean, SD)	1.2 (0.6)	1.3 (1.2)	
	Day 4 (mean, SD)	1.1 (0.5)	1.1 (0.6)	
	Percentage of change (from baseline value, %)	-8.3	-15.4	**0.007**
**INR**	Baseline (mean, SD)	1.3 (0.3)	1.5 (0.5)	
	Day 4 (mean, SD)	1.2 (0.3)	1.4 (0.3)	
	Percentage of change (from baseline value, %)	-7.7	-6.7	0.250
**Platelets (10**^**3**^ **cells/**μ**L)**	Baseline (mean, SD)	229.8 (109.9)	182.4 (92.8)	
	Day 4 (mean, SD)	200.1 (97.5)	175.4 (78.2)	
	Percentage of change (from baseline value, %)	-12.9	-3.8	0.111
**Hemoglobin (g/dL)**	Baseline (mean, SD)	11.9 (2.2)	12.8 (2.8)	
	Day 4 (mean, SD)	11.3 (2.1)	12.0 (2.4)	
	Percentage of change (from baseline value, %)	-5.0	-6.3	0.451
**Serum sodium (mEq/L)**	Baseline (mean, SD)	136.5 (3.8)	138.2 (3.8)	
	Day 4 (mean, SD)	137.5 (3.2)	138.9 (3.9)	
	Percentage of change (from baseline value, %)	+0.7	+0.5	0.288

Patients with missing data on day 4 were excluded from this analysis.

**Fig 2:**
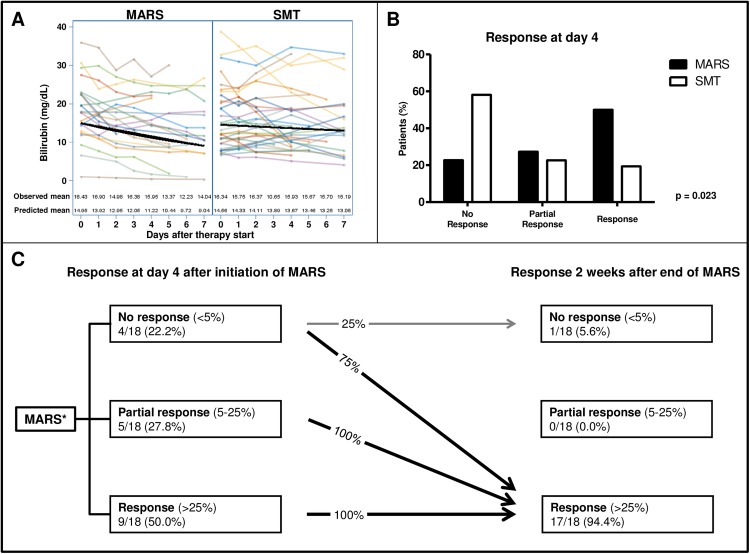
Patients with acute liver injury: Course of bilirubin values and response to MARS treatment in the short term and 2 weeks after the end of MARS therapy. (A) Course of bilirubin values during the first week of therapy (MARS: 22 patients, SMT: 31 patients). (B) Response classification at day 4 of therapy (No response *vs*. Partial response *vs*. Response) was based on the calculated bilirubin reduction and compared to the bilirubin baseline value. (C) Corresponding response classification two weeks after the last MARS session. * Patients with liver transplantation or missing data at follow-up or those who had died were excluded from this analysis (in total, 4 patients).

Regarding acute liver injury, bilirubin decline within in the first week of therapy was significantly more pronounced in the MARS group than in the SMT group (reduction of bilirubin per day: -1.07 mg/dL (95%CI: -1.11, -1.03) vs. -0.98 mg/dL (95%CI: -1.07, -0.91), p = 0.0144) (**Fig 2A**). This difference was also evident after adopting response criteria. Specifically, more patients were classified as responders (R) at day 4 in the MARS group than in the SMT group. In terms of acute liver injury patients receiving MARS therapy, 50% showed a full response (R) whereas 19.4% of patients in the SMT group showed a full response (**Fig 2B**). By contrast, only 22.7% of patients were non-responders after MARS therapy (vs. 58.1% with SMT). Remarkably, all responders after the short-term evaluation remained responders two weeks after finishing MARS therapy (**Fig 2C**). Furthermore, nearly all of the MARS patients with no or partial initial/short-term response (classified as NR or PR) developed a sustained drop in bilirubin values after the termination of MARS therapy. In summary, with the exception of one patient, all patients with acute liver injury receiving MARS therapy showed a sustained decrease in bilirubin values.

**Fig 3:**
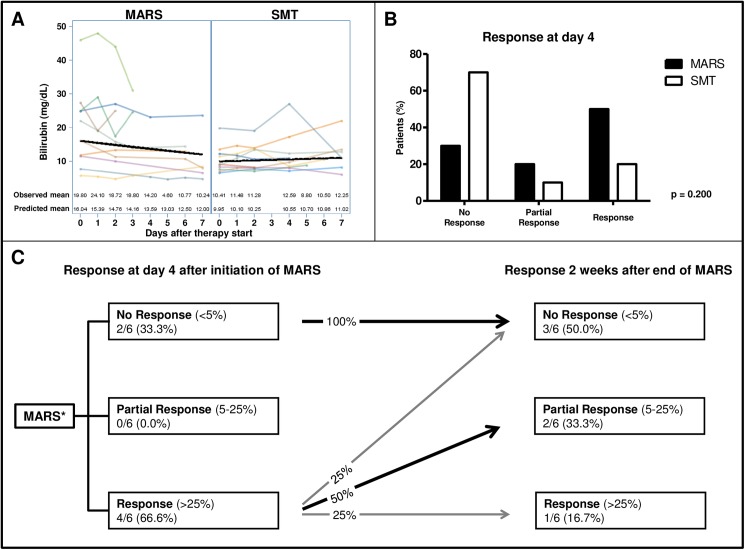
Patients with graft dysfunction: Course of bilirubin values and response to MARS treatment in the short term and 2 weeks after the end of MARS therapy. (A) Course of bilirubin values during the first week of therapy (MARS: 10 patients, SMT: 10 patients). (B) Response classification at day 4 of therapy (No response *vs*. Partial response *vs*. Response) was based on the calculated bilirubin reduction and compared to the bilirubin baseline value. (C) Corresponding response classification two weeks after the last MARS session. * Patients with liver transplantation or missing data at follow-up or those who had died were excluded from this analysis (in total, 4 patients).

In patients with GD, the decrease in bilirubin values was more pronounced in the MARS group than in the SMT group; however, there was not a statistically significant difference (MARS: reduction of bilirubin per day of -1.04 mg/dL (95%CI: -1.08, -1.00); SMT: increase of bilirubin per day of +1.01 mg/dL (95%CI: -0.92, +1.12); p = 0.0538) (**Fig 3A**). Similar to the classification of the patients with acute liver injury, the classification of the patients with GD according to the response criteria showed a trend toward a better short-term response in the MARS group than in the SMT group (**Fig 3B**). However, many initial responders (75%) worsened in terms of bilirubin values two weeks after the termination of therapy (**Fig 3C**). Notably, all non-responders remained non-responders (100%).

## Discussion

This study analyzed the effects of MARS therapy in two patient groups, patients with acute liver injury and patients with GD. In both groups, MARS showed no advantage over SMT with regard to 28-day mortality. However, analyses of bilirubin dynamics and of response to therapy revealed significant differences. By excluding patients suffering from extrahepatic origin of liver injury we intended to focus on diseases with primary liver impairment. However MARS therapy may also have beneficial effects on symptoms of liver disease secondary to other causes. In the acute liver injury group, compared with SMT, MARS therapy resulted in a higher percentage of patients with a short-term reduction of bilirubin values. A rapid response to MARS was predictive of a sustained response after the end of ALS treatment; thus, MARS could have a catalytic function in the course of this disease. Initial bilirubin values were higher in the MARS group compared to the SMT group. Thus it is unknown whether differences of baseline bilirubin values might have influence on the course of therapy response.

These findings were supported by other studies that also reported a stabilization of liver function and an improvement in liver regeneration through the use of the MARS system for treating ALF [12,19,20]. In contrast to chronic liver failure, the native liver often shows considerable regeneration potential due to the absence of an underlying liver disease with preexisting organ damage [21].

The findings for the GD group were different. A significant increase in the short-term response after MARS treatment was also observed (compared to that after SMT); however, in some patients, this effect lasted for a short period of time, which led to the conclusion that the effects of MARS treatment were not persistent. Moreover, no further spontaneous response after the termination of therapy was observed. Therefore, the response to MARS therapy could be hypothesized to predict the chance of liver recovery in this entity. In addition, an amelioration of kidney function parameters was observed after MARS therapy in this subgroup. This is especially notable considering that these patients had higher initial bilirubin values compared to patients receiving SMT only.

A comparison between our cohort and patients from other studies analyzing ALS illustrated significant differences in patient characteristics [10–12,14,15].

All patients with drug-induced liver injury analyzed in our study predominantly displayed disease that was moderate to severe [22]. Compared to patients in other studies, our patients were significantly healthier because none presented with a simultaneous appearance of HE or coagulation defects or fulfilled the criteria for ALF [23,24]. Although positive effects of MARS treatment on HE are known, we did not further analyze this association because all patients included in this study had low or no HE.

The findings of our study emphasized the need for more precise patient selection prior to therapy, as the various liver diseases with different severities show specific response characteristics to ALS therapy.

We found MARS therapy to exert a catalytic function in patients with liver injury and without secondary organ failure, such as HE or coagulation failure, among others. In addition, MARS could support (spontaneous) regeneration but most likely has no effect on mortality.

However, in patients with multiorgan failure, the therapeutic effects of ALS treatment are more essential and result in positive effects on survival [19,25], which leads to the hypothesis that (only) ALF patients with a high MELD score or multiorgan failure could benefit from MARS therapy. This hypothesis is in accordance with the results of a recently published study by Larsen et al. using a different ALS method. They reported on critically ill ALF patients who were treated with high volume plasma exchange to correct the patients’ blood composition [26]. The positive effects on survival observed in this study suggested that early ALS application has the ability to modulate the pro-inflammatory “storm” and therefore limit the anti-inflammatory response.

Analogous to acute liver injury, the benefits of therapy in ACLF depend on the incidence of multiorgan failure (MELD score > 30 or ACLF grade > = 2)[5,27]. In general, an increased ACLF grade is associated with a lower probability of the spontaneous improvement and resolution of ACLF [28]. These patients especially require extensive clinical resources and could benefit from MARS dialysis.

What is the significance of these results in terms of patient management? We believe that our findings are very important for optimizing individual patient allocation criteria to MARS therapy and for generating new hypotheses for the design of future studies. Considering results from previous studies, we hypothesized that the properties of MARS therapy, including detoxification and metabolic stabilization, should be considered especially in patients with rapid dynamics transitioning to multiorgan failure and in those with existing multiorgan failure. MARS therapy appears to have its most advantageous impact in this high-risk population. By contrast, a hepatic injury without transition into multiorgan failure or a low MELD score does not appear to produce a benefit in terms of mortality, as shown in our cohort.

Certain limitations of the current study warrant discussion. First, the design of this study is retrospective, and there were a limited number of patients in each subgroup, which also prohibits more differentiated evaluations of specific subgroups. For example, etiology may have an impact on response and outcome but could not be analyzed without being underpowered. The imbalance of viral/ autoimmune caused liver injury with predominance in the SMT group might potentially have also biased the comparison between MARS and SMT.

Although prospective randomized trials are needed to show a positive outcome of MARS on survival, they are difficult to be performed for several reasons, including rarity, severity and heterogeneity of the disease as well as varying access (modalities) to transplantation between regions and countries. The fact that the effects of MARS therapy compared to the SMT in GD were shown for the first time should not reduce the need for prospective studies with a larger number of subjects.

Although it did not improve 28-day mortality, MARS therapy enhanced the short-term response in patients with acute liver injury as well as in those with GD. Especially in cases of acute hepatic injury, the use of MARS therapy resulted in the sustained stabilization of the liver function and improved liver regeneration. However, this persistent response to therapy was not distinct in GD. The results of the present study improve the understanding of MARS therapy in two different etiologies. Additionally, the short-term response to MARS could be hypothesized to predict the future course of the disease.

## Supporting information

S1 Table Baseline patient characteristics of patients with severe hepatitis and liver graft dysfunction: Comparison of MARS and standard medical treatment (SMT). Abbr.: MAP: Mean arterial pressure; BUN: blood urea nitrogen; INR: international normalized ratio; MELD: model of end-stage liver disease; AST: aspartate transaminase; ALT: alanine transaminase.
(DOCX)

S2 Table Short-term response of laboratory parameters. Patients with missing data on day 4 were excluded from this analysis.
(DOCX)
