# Supplementary material for: Molecular adsorbent recirculating system (MARS) in acute liver injury and graft dysfunction: Results from a case-control study
Source: PLoS One. 2017 Apr 12;12(4):e0175529. doi: 10.1371/journal.pone.0175529 (PMC5389829; doi:10.1371/journal.pone.0175529)
Supplement: S1 Table — Abbr.: MAP: Mean arterial pressure; BUN: blood urea nitrogen; INR: international normalized ratio; MELD: model of end-stage liver disease; AST: aspartate transaminase; ALT: alanine transaminase. (DOCX) [file pone.0175529.s001.docx]

**S1 Table. Baseline patient characteristics of patients with acute liver injury and liver graft dysfunction: Comparison of MARS and standard medical treatment (SMT).**

|  | **Graft dysfunction** | |  | **Acute liver injury** | |  |
| --- | --- | --- | --- | --- | --- | --- |
| Laboratory Parameter | **MARS**  **(n = 10)** | **SMT**  **(n = 10)** | **p-value** | **MARS**  **(n = 22)** | **SMT**  **(n = 31)** | **p-value** |
| Age (years), mean (SD) | 50.7 (13.8) | 49.4 (12.7) | 0.684 | 48.1 (19.6) | 49.4 (17.1) | 0.814 |
| Male sex, n (%) | 8 (80) | 6 (60) | 0.329 | 12 (54.5) | 17 (54.8) | 0.983 |
| Body weight (kg), mean (SD) | 80.3 (15.6) | 74.7 (23.1) | 0.573 | 82.9 (19.6) | 76.3 (21.4) | 0.278 |
| Hepatic encephalopathy, n (%)  Grade ≤1  Grade 2-3 | 10 (100)  0 | 10 (100)  0 | 0.606 | 22 (100)  1 (4.8) | 28 (90.3)  3 (9.7) | 0.532 |
| MAP (mmHg), mean (SD) | 92.9 (10.4) | 87.6 (15.0) | 0.195 | 99.9 (11.6) | 102.3 (13.1) | 0.521 |
| Heart rate (bpm), mean (SD) | 78.2 (10.6) | 79.6 (14.8) | 1.000 | 74.0 (7.0) | 83.3 (15.2) | 0.174 |
| Laboratory data, mean (SD)  Total bilirubin (mg/dL)  AST (U/L)  ALT (U/L)  Serum sodium (mEq/L)  Serum potassium (mEq/L)  Creatinine (mg/dL)  BUN (mg/dL)  White blood count (10^3^ cells/µL)  Hemoglobin (g/dL)  Platelets (10^3^ cells/µL)  Albumin (g/dL)  Prothrombin level (Quick) (%)  INR | 19.8 (11.9)  142.0 (65.7)  124.5 (96.8)  136.5 (3.4)  4.1 (0.6)  1.7 (0.6)  38.1 (19.7)  7.8 (4.0)  9.9 (1.7)  193.4 (110.0)  3.3 (0.4)  85.2 (18.2)  1.1 (0.1) | 10.4 (4.0)  413.5 (734.1)  187.6 (278.6)  135.4 (3.2)  4.1 (0.5)  1.5 (0.5)  28.7 (12.2)  5.4 (3.3)  10.1 (3.6)  163.7 (102.8)  3.1 (0.8)  57.7 (27.8)  1.7 (0.7) | 0.052  0.393  0.684  0.515  0.696  0.631  0.190  0.182  0.815  0.604  1.000  **0.035**  **0.013** | 18.4 (8.1)  784.7 (804.3)  993.4 (879.6)  136.5 (4.0)  4.0 (0.4)  1.0 (0.5)  17.8 (12.9)  7.7 (3.0)  12.7 (1.9)  246.4 (108.2)  3.5 (0.6)  67.0 (28.6)  1.4 (0.4) | 16.3 (8.5)  1293.8 (871.6)  1810.3 (1249.3)  139.2 (3.5)  3.8 (0.5)  1.3 (1.3)  17.1 (13.3)  7.7 (2.6)  13.6 (1.9)  187.8 (90.7)  3.2 (0.4)  56.0 (18.1)  1.5 (0.4) | 0.230  **0.021**  **0.026**  **0.026**  0.163  0.658  0.680  0.807  0.068  0.056  0.667  0.263  0.340 |
| MELD Score, mean (SD) | 20.3 (7.9) | 24.0 (5.2) | 0.353 | 20.7 (6.9) | 22.0 (4.7) | 0.814 |

Abbr.: MAP: Mean arterial pressure; BUN: blood urea nitrogen; INR: international normalized ratio; MELD: model of end-stage liver disease; SD: standard deviation; AST: aspartate transaminase; ALT: alanine transaminase
