# Supplementary material for: Molecular adsorbent recirculating system (MARS) in acute liver injury and graft dysfunction: Results from a case-control study
Source: PLoS One. 2017 Apr 12;12(4):e0175529. doi: 10.1371/journal.pone.0175529 (PMC5389829; doi:10.1371/journal.pone.0175529)
Supplement: S2 Table — Patients with missing data on day 4 were excluded from this analysis. (DOCX) [file pone.0175529.s002.docx]

**S2 Table. Short-term response of laboratory parameters.**

|  | **Graft dysfunction** | | |  | **Acute liver injury** | |  |
| --- | --- | --- | --- | --- | --- | --- | --- |
| **Laboratory Parameter** | **MARS** | **SMT** | | **p-value** | **MARS** | **SMT** | **p-value** |
| Bilirubin (mg/dL)  Baseline (mean, SD)  Day 4 (mean, SD)  Percentage of change (from baseline value, %) | 19.8 (11.9)  16.1 (9.1)  -18.7 | | 10.4 (4.0)  11.5 (6.3)  +10.6 | 0.063 | 18.4 (8.1)  14.0 (7.6)  -23.9 | 16.3 (8.5)  15.3 (8.2)  -6.1 | **0.005** |
| Creatinine (mg/dL)  Baseline (mean, SD)  Day 4 (mean, SD)  Percentage of change (from baseline value, %) | 1.7 (0.6)  1.3 (0.5)  -23.5 | | 1.5 (0.5)  1.3 (0.4)  -13.3 | 0.237 | 1.0 (0.5)  1.0 (0.5)  ±0 | 1.3 (1.3)  1.0 (0.7)  -23.1 | **0.001** |
| INR  Baseline (mean, SD)  Day 4 (mean, SD)  Percentage of change (from baseline value, %) | 1.1 (0.1)  1.1 (0.1)  ±0 | | 1.7 (0.7)  1.5 (0.4)  -11.8 | 0.968 | 1.4 (0.4)  1.3 (0.3)  -7.1 | 1.5 (0.4)  1.4 (0.3)  -6.7 | 0.370 |
| Platelets (10^3^ cells/µL)  Baseline (mean, SD)  Day 4 (mean, SD)  Percentage of change (from baseline value, %) | 193.4 (110.0)  177.4 (94.2)  -8.3 | | 163.7 (102.8)  143.4 (80.3)  -12.4 | 0.139 | 246.4 (108.2)  209.8 (99.6)  -14.9 | 187.8 (90.7)  186.5 (75.8)  -0.7 | 0.300 |
| Hemoglobin (g/dL)  Baseline (mean, SD)  Day 4 (mean, SD)  Percentage of change (from baseline value, %) | 9.8 (1.7)  9.4 (1.8)  -4.1 | | 10.1 (3.4)  10.1 (2.69)  ±0 | 0.408 | 12.7 (1.9)  11.9 (1.91)  -6.3 | 13.6 (1.9)  12.6 (2.0)  -7.4 | 0.271 |
| Serum sodium (mEq/L)  Baseline (mean, SD)  Day 4 (mean, SD)  Percentage of change (from baseline value, %) | 136.5 (3.4)  135.7 (3.9)  -0.6 | | 135.4 (3.2)  135.7 (4.1)  +0.2 | 0.274 | 136.5 (4.0)  138.1 (2.8)  -1.2 | 139.2 (3.5)  140.0 (3.3)  +0.6 | 0.086 |

Abbr.: SD: standard deviation; INR: international normalized ratio

Patients with missing data on day 4 were excluded from this analysis.
